# Crystal structure of 3-[4-(1-methyl­eth­yl)phen­yl]-1-(naphthalen-2-yl)prop-2-en-1-one

**DOI:** 10.1107/S1600536814017528

**Published:** 2014-08-16

**Authors:** Sid Assia, Messai Amel, Ziani Nouara, Mokhtari Mahieddine, Lamara Kaddour

**Affiliations:** a Laboratoire de Chimie Appliqué, et Matériaux Téchnologique, Université Larbi Ben M’Hidi, 04000 Oum El Bouaghi, Algeria; b Laboratoire des Structures, Propriétés et Interactions InterAtomiques, Université Abbes Laghrour Khenchela, 40000 Khenchela, Algeria

**Keywords:** crystal structure, chalcones, prop-2-en-1-one, Claisen–Schmidt, aldolic condensation

## Abstract

The title compound, C_22_H_20_O, was synthesized by reacting 4-iso­propyl­benzaldehyde with 2-acetonaphtone by aldolic condensation under Claisen–Schmidt conditions. The mol­ecule consists of a naphthalene group and a benzene ring with a pendant isopropyl moiety, both rings bound by a propenone linker. The naphthalene ring system is almost planar [maximum deviation from the least-squares plane = 0.026 (10) Å] and subtends a dihedral angle of 52.31 (4)° with the benzene ring. The propenone linker, in turn, deviates slightly more from planarity [maximum deviation = 0.125 (18) Å] and has its least-squares plane oriented midway the former two, at 25.62 (6) and 28.02 (5)° from the naphthalene ring system and the benzene ring, respectively. Finally, the isopropyl group presents its CC_2_ plane almost perpendicular to the benzene ring, at 85.30 (4)°. No significant hydrogen bonding or π–π stacking inter­actions are found in the crystal structure.

## Related literature   

For chalcones as important starting materials or inter­mediates for the synthesis of naturally occurring flavonoids, see: Geissmann (1962 ▶); Mabry *et al.* (1970 ▶); Harborne (1988 ▶, 1994 ▶); Wong (1970 ▶). For compilation and discussion of the syntheses of chalcones and their analogues, see: Dhar (1981 ▶); Lévai (1997 ▶).
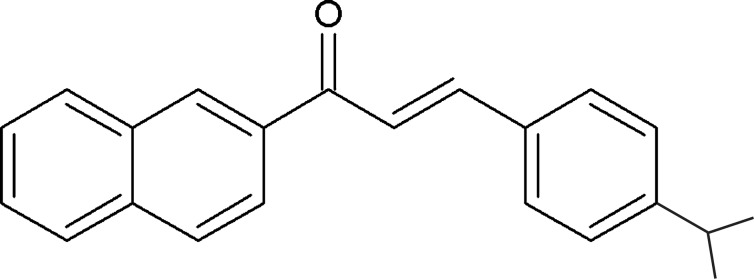


## Experimental   

### Crystal data  

C_22_H_20_O
*M* *_r_* = 300.40
Monoclinic, 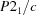

*a* = 5.8326 (2) Å
*b* = 17.8578 (6) Å
*c* = 15.6469 (5) Å
β = 91.136 (3)°
*V* = 1629.42 (9) Å^3^
*Z* = 4
Cu *K*α radiation
μ = 0.56 mm^−1^
*T* = 150 K
0.60 × 0.17 × 0.17 mm

### Data collection  

Agilent Xcalibur (Atlas, Gemini ultra) diffractometer
Absorption correction: multi-scan (*CrysAlis PRO*; Agilent, 2013 ▶) *T*_min_ = 0.794, *T*_max_ = 1.000
12851 measured reflections
2871 independent reflections
2659 reflections with *I* > 2s*I*)
*R*_int_ = 0.035

### Refinement  

*R*[*F* ^2^ > 2σ(*F* ^2^)] = 0.035
*wR*(*F* ^2^) = 0.097
*S* = 1.04
2871 reflections
208 parameters
H-atom parameters constrained
Δρ_max_ = 0.19 e Å^−3^
Δρ_min_ = −0.20 e Å^−3^

### 

Data collection: *CrysAlis PRO* (Agilent, 2013 ▶); cell refinement: *CrysAlis PRO*; data reduction: *CrysAlis PRO*; program(s) used to solve structure: *SIR2004* (Altomare *et al.*, 1999 ▶); program(s) used to refine structure: *SHELXL97* (Sheldrick, 2008 ▶); molecular graphics: *ORTEP-3 for Windows* (Farrugia, 2012 ▶) and *PLATON* (Spek, 2009 ▶); software used to prepare material for publication: *WinGX* (Farrugia, 2012 ▶).

## Supplementary Material

Crystal structure: contains datablock(s) I. DOI: 10.1107/S1600536814017528/bg2534sup1.cif

Structure factors: contains datablock(s) I. DOI: 10.1107/S1600536814017528/bg2534Isup2.hkl

Supporting information file. DOI: 10.1107/S1600536814017528/bg2534Isup3.cml

**Fig. 1.**
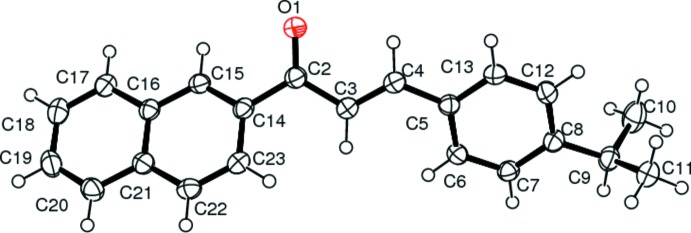
The title compound with displacement ellipsoids drawn at the 50% probability level.

. DOI: 10.1107/S1600536814017528/bg2534fig1.tif
The title compound with displacement ellipsoids drawn at the 50% probability level.

CCDC reference: 1017044

Additional supporting information:  crystallographic information; 3D view; checkCIF report

## supplementary crystallographic information

### S6. Refinement

Crystal data, data collection and structure refinement details are summarized in Table 1.

### S7. Comment

Chalcones are versatile and convenient intermediates for the synthesis of a wide variety of heterocyclic compounds. The enone moiety of the molecule is a favourable unit for dipolar cycloaddition with numerous reagents providing heterocyclic compounds of different ring sizes with one or several heteroatoms. Their reactions with dinucleophiles usually result in the formation of polycyclic ring systems which may be the skeleton of important heterocyclic compounds.

Among the chalcones and their analogues are especially important starting materials or intermediates for the synthesis of naturally occurring flavonoids (Geissmann, 1962; Mabry *et al.*,1970; Harborne, 1988,1994; Wong, 1970) and various nitrogen-containing heterocyclic compounds. For this reason, their syntheses have been compiled and discussed in various accounts (Dhar *et al.*, 1981; Lévai, 1997).

We report here the synthesis and the crystal structure determination of 3-[4-(1-methylethyl)phenyl]-1-(2-naphthalenyl)- 2-Propen-1-one (I). The title compound, C_22_H_20_O, was synthesized by reacting 4-isopropyl benzaldehyde with 2-acetonaphtone by aldolic condensation using Claisen-Schmidth conditions. The molecule consist basically of a naphthalene group, a benzene ring with a pendant isopropyl moiety, both rings bound by a propenone linker. The naphthalene and benzene rings are planar (maximum deviations from their L·S. planes: 0.026 (10) and 0.0148 (6) Å, respectively) subtending an angle of 52.31 (4)°. The propenone linker, in turn, deviates slightly more from planarityly (max.dev; 0.125 Å) and has its l.s. plane oriented midway the former two, at 25.62 (6) and 28.02 (5)° from each one, respectively. Finally, the isopropyl group presents its CC_2_ plane almost perpendicular to the benzene ring, at 85.30 (4)°. No significant hydrogen bonding nor π–π stacking interactions are found in the crystal structure.

### S8. Experimental

A mixture of 2-acetonaphtone (0.01 mole) and 4-isopropyl benzaldehyde (0.01 mole) was stirred in ethanol (50 ml) and then a solution of 15 ml sodium hydroxide (0.04 mole) was added drop wase. The mixture was kept for four h at room temperature and then it was poured into crushed ice and acidified with dil. HCl. The product precipitates out as solid. Then it was filtered. Single yellow crystals of 3-[4-(1-methylethyl)phenyl]-1-(2-naphthalenyl)- 2-Propen-1-one were obtain after crystallized from ethyl acetate with 76% in yield.

### S9. Refinement

H atoms were all located in a difference map, repositioned geometrically and further refined with riding constraints (C—H in the range 0.93–0.98 Å) and *U*_iso_(H) (in the range 1.2–1.5 times *U*_eq_ of the parent atom)

### Crystal data

**Table:**

C_22_H_20_O	*F*(000) = 640
*M**_r_* = 300.40	*D*_x_ = 1.225 Mg m^−^^3^
Monoclinic, *P*2_1_/*c*	Cu *K*α radiation, λ = 1.54180 Å
Hall symbol: -P 2ybc	Cell parameters from 7372 reflections
*a* = 5.8326 (2) Å	θ = 4.9–66.8°
*b* = 17.8578 (6) Å	µ = 0.56 mm^−^^1^
*c* = 15.6469 (5) Å	*T* = 150 K
β = 91.136 (3)°	Needle, colorless
*V* = 1629.42 (9) Å^3^	0.60 × 0.17 × 0.17 mm
*Z* = 4	

### Data collection

**Table:**

Agilent Xcalibur (Atlas, Gemini ultra) diffractometer	2871 independent reflections
Radiation source: Enhance Ultra (Cu) X-ray Source	2659 reflections with *I* > 2s˘*I*)
Mirror monochromator	*R*_int_ = 0.035
Detector resolution: 10.4678 pixels mm^-1^	θ_max_ = 67.8°, θ_min_ = 3.8°
ω scans	*h* = −6→6
Absorption correction: multi-scan (*CrysAlis PRO*; Agilent, 2013)	*k* = −21→20
*T*_min_ = 0.794, *T*_max_ = 1.000	*l* = −18→18
12851 measured reflections	

### Refinement

**Table:**

Refinement on *F*^2^	Primary atom site location: structure-invariant direct methods
Least-squares matrix: full	Secondary atom site location: difference Fourier map
*R*[*F*^2^ > 2σ(*F*^2^)] = 0.035	Hydrogen site location: inferred from neighbouring sites
*wR*(*F*^2^) = 0.097	H-atom parameters constrained
*S* = 1.04	*w* = 1/[σ^2^(*F*_o_^2^) + (0.0512*P*)^2^ + 0.3775*P*] where *P* = (*F*_o_^2^ + 2*F*_c_^2^)/3
2871 reflections	(Δ/σ)_max_ < 0.001
208 parameters	Δρ_max_ = 0.19 e Å^−^^3^
0 restraints	Δρ_min_ = −0.20 e Å^−^^3^

### Special details

**Table:**

Geometry. All e.s.d.'s (except the e.s.d. in the dihedral angle between two l.s. planes) are estimated using the full covariance matrix. The cell e.s.d.'s are taken into account individually in the estimation of e.s.d.'s in distances, angles and torsion angles; correlations between e.s.d.'s in cell parameters are only used when they are defined by crystal symmetry. An approximate (isotropic) treatment of cell e.s.d.'s is used for estimating e.s.d.'s involving l.s. planes.
Refinement. Refinement of *F*^2^ against ALL reflections. The weighted *R*-factor *wR* and goodness of fit *S* are based on *F*^2^, conventional *R*-factors *R* are based on *F*, with *F* set to zero for negative *F*^2^. The threshold expression of *F*^2^ > σ(*F*^2^) is used only for calculating *R*-factors(gt) *etc*. and is not relevant to the choice of reflections for refinement. *R*-factors based on *F*^2^ are statistically about twice as large as those based on *F*, and *R*- factors based on ALL data will be even larger.

### Fractional atomic coordinates and isotropic or equivalent isotropic displacement parameters (Å^2^)

**Table:**

	*x*	*y*	*z*	*U*_iso_*/*U*_eq_	
O1	1.18162 (14)	0.47189 (5)	0.89162 (6)	0.0419 (2)	
C2	0.97778 (19)	0.48838 (6)	0.89245 (7)	0.0303 (3)	
C3	0.79779 (19)	0.43015 (6)	0.89363 (7)	0.0299 (3)	
H3A	0.6454	0.4433	0.8835	0.036*	
C4	0.85268 (19)	0.35895 (6)	0.90914 (7)	0.0288 (3)	
H4	1.0034	0.3498	0.9266	0.035*	
C5	0.70261 (18)	0.29353 (6)	0.90177 (7)	0.0269 (2)	
C6	0.48502 (19)	0.29638 (6)	0.86111 (7)	0.0286 (2)	
H6	0.4267	0.3421	0.8424	0.034*	
C7	0.35703 (19)	0.23184 (6)	0.84868 (7)	0.0293 (3)	
H7	0.2147	0.2349	0.8211	0.035*	
C8	0.43772 (19)	0.16242 (6)	0.87684 (7)	0.0300 (3)	
C9	0.3002 (2)	0.09158 (7)	0.85949 (8)	0.0348 (3)	
H9	0.1649	0.1054	0.8249	0.042*	
C10	0.4352 (3)	0.03475 (8)	0.80940 (9)	0.0503 (4)	
H10A	0.4867	0.0572	0.7575	0.075*	
H10B	0.3391	−0.0075	0.7959	0.075*	
H10C	0.5653	0.0184	0.8430	0.075*	
C11	0.2174 (2)	0.05676 (7)	0.94176 (9)	0.0398 (3)	
H11A	0.1308	0.0930	0.9729	0.060*	
H11B	0.3470	0.0408	0.9759	0.060*	
H11C	0.1220	0.0144	0.9285	0.060*	
C12	0.6506 (2)	0.16017 (6)	0.92011 (8)	0.0337 (3)	
H12	0.7051	0.1148	0.9413	0.040*	
C13	0.78131 (19)	0.22435 (6)	0.93182 (7)	0.0312 (3)	
H13	0.9229	0.2213	0.9600	0.037*	
C14	0.90716 (18)	0.56875 (6)	0.88921 (7)	0.0276 (3)	
C15	1.05481 (18)	0.61962 (6)	0.85525 (7)	0.0275 (2)	
H15	1.1945	0.6031	0.8347	0.033*	
C16	1.00104 (18)	0.69660 (6)	0.85048 (7)	0.0271 (2)	
C17	1.1488 (2)	0.74932 (6)	0.81351 (7)	0.0320 (3)	
H17	1.2870	0.7334	0.7910	0.038*	
C18	1.0921 (2)	0.82339 (7)	0.81026 (8)	0.0371 (3)	
H18	1.1918	0.8575	0.7858	0.045*	
C19	0.8844 (2)	0.84836 (7)	0.84361 (8)	0.0392 (3)	
H19	0.8469	0.8989	0.8411	0.047*	
C20	0.7377 (2)	0.79899 (7)	0.87967 (8)	0.0354 (3)	
H20	0.6007	0.8162	0.9019	0.042*	
C21	0.79051 (19)	0.72157 (6)	0.88392 (7)	0.0289 (3)	
C22	0.64154 (19)	0.66816 (7)	0.91904 (7)	0.0312 (3)	
H22	0.5034	0.6840	0.9416	0.037*	
C23	0.69527 (19)	0.59380 (6)	0.92065 (7)	0.0303 (3)	
H23	0.5919	0.5595	0.9426	0.036*	

### Atomic displacement parameters (Å^2^)

**Table:**

	*U*^11^	*U*^22^	*U*^33^	*U*^12^	*U*^13^	*U*^23^
O1	0.0268 (5)	0.0322 (5)	0.0666 (6)	0.0005 (3)	0.0017 (4)	0.0070 (4)
C2	0.0276 (6)	0.0309 (6)	0.0323 (6)	−0.0010 (5)	−0.0008 (4)	0.0019 (5)
C3	0.0257 (6)	0.0300 (6)	0.0340 (6)	−0.0014 (4)	−0.0001 (4)	0.0000 (4)
C4	0.0255 (5)	0.0315 (6)	0.0295 (6)	−0.0010 (4)	0.0004 (4)	−0.0009 (4)
C5	0.0259 (6)	0.0272 (5)	0.0276 (5)	−0.0001 (4)	0.0034 (4)	−0.0007 (4)
C6	0.0281 (6)	0.0269 (5)	0.0310 (6)	0.0024 (4)	0.0004 (4)	0.0021 (4)
C7	0.0247 (5)	0.0327 (6)	0.0304 (6)	−0.0010 (4)	−0.0016 (4)	0.0020 (4)
C8	0.0297 (6)	0.0300 (6)	0.0304 (6)	−0.0036 (4)	0.0013 (4)	0.0023 (4)
C9	0.0347 (6)	0.0309 (6)	0.0387 (6)	−0.0067 (5)	−0.0058 (5)	0.0042 (5)
C10	0.0622 (9)	0.0414 (7)	0.0477 (8)	−0.0157 (6)	0.0102 (7)	−0.0098 (6)
C11	0.0374 (7)	0.0346 (6)	0.0476 (7)	−0.0070 (5)	0.0038 (5)	0.0040 (5)
C12	0.0325 (6)	0.0270 (6)	0.0414 (7)	0.0000 (5)	−0.0036 (5)	0.0062 (5)
C13	0.0248 (6)	0.0322 (6)	0.0364 (6)	0.0006 (4)	−0.0031 (4)	0.0027 (5)
C14	0.0259 (5)	0.0289 (6)	0.0281 (5)	−0.0029 (4)	−0.0025 (4)	−0.0001 (4)
C15	0.0235 (5)	0.0304 (6)	0.0286 (5)	0.0000 (4)	−0.0005 (4)	−0.0018 (4)
C16	0.0267 (6)	0.0287 (6)	0.0257 (5)	−0.0021 (4)	−0.0032 (4)	−0.0012 (4)
C17	0.0305 (6)	0.0325 (6)	0.0328 (6)	−0.0038 (5)	−0.0008 (5)	0.0008 (5)
C18	0.0422 (7)	0.0309 (6)	0.0380 (6)	−0.0081 (5)	−0.0029 (5)	0.0033 (5)
C19	0.0471 (7)	0.0260 (6)	0.0440 (7)	0.0010 (5)	−0.0070 (6)	−0.0028 (5)
C20	0.0351 (6)	0.0325 (6)	0.0383 (6)	0.0038 (5)	−0.0031 (5)	−0.0078 (5)
C21	0.0287 (6)	0.0306 (6)	0.0272 (5)	−0.0002 (4)	−0.0040 (4)	−0.0035 (4)
C22	0.0261 (6)	0.0374 (6)	0.0304 (6)	0.0008 (5)	0.0024 (4)	−0.0036 (5)
C23	0.0267 (6)	0.0330 (6)	0.0311 (6)	−0.0044 (4)	0.0013 (4)	0.0010 (5)

### Geometric parameters (Å, º)

**Table:**

O1—C2	1.2252 (14)	C11—H11C	0.9600
C2—C3	1.4779 (16)	C12—C13	1.3865 (16)
C2—C14	1.4938 (16)	C12—H12	0.9300
C3—C4	1.3323 (16)	C13—H13	0.9300
C3—H3A	0.9300	C14—C15	1.3666 (16)
C4—C5	1.4630 (15)	C14—C23	1.4119 (16)
C4—H4	0.9300	C15—C16	1.4116 (16)
C5—C13	1.3962 (16)	C15—H15	0.9300
C5—C6	1.4093 (16)	C16—C17	1.4085 (16)
C6—C7	1.3848 (16)	C16—C21	1.4163 (16)
C6—H6	0.9300	C17—C18	1.3643 (17)
C7—C8	1.3944 (16)	C17—H17	0.9300
C7—H7	0.9300	C18—C19	1.4011 (19)
C8—C12	1.4031 (17)	C18—H18	0.9300
C8—C9	1.5192 (15)	C19—C20	1.3592 (18)
C9—C10	1.5126 (19)	C19—H19	0.9300
C9—C11	1.5173 (17)	C20—C21	1.4177 (16)
C9—H9	0.9800	C20—H20	0.9300
C10—H10A	0.9600	C21—C22	1.4091 (16)
C10—H10B	0.9600	C22—C23	1.3645 (17)
C10—H10C	0.9600	C22—H22	0.9300
C11—H11A	0.9600	C23—H23	0.9300
C11—H11B	0.9600		
			
O1—C2—C3	121.38 (10)	H11B—C11—H11C	109.5
O1—C2—C14	119.84 (10)	C13—C12—C8	121.27 (11)
C3—C2—C14	118.75 (10)	C13—C12—H12	119.4
C4—C3—C2	120.37 (10)	C8—C12—H12	119.4
C4—C3—H3A	119.8	C12—C13—C5	120.71 (10)
C2—C3—H3A	119.8	C12—C13—H13	119.6
C3—C4—C5	127.34 (11)	C5—C13—H13	119.6
C3—C4—H4	116.3	C15—C14—C23	119.18 (10)
C5—C4—H4	116.3	C15—C14—C2	118.46 (10)
C13—C5—C6	118.11 (10)	C23—C14—C2	122.36 (10)
C13—C5—C4	119.20 (10)	C14—C15—C16	121.79 (10)
C6—C5—C4	122.60 (10)	C14—C15—H15	119.1
C7—C6—C5	120.76 (10)	C16—C15—H15	119.1
C7—C6—H6	119.6	C17—C16—C15	122.37 (10)
C5—C6—H6	119.6	C17—C16—C21	118.91 (10)
C6—C7—C8	121.20 (10)	C15—C16—C21	118.72 (10)
C6—C7—H7	119.4	C18—C17—C16	120.89 (11)
C8—C7—H7	119.4	C18—C17—H17	119.6
C7—C8—C12	117.89 (10)	C16—C17—H17	119.6
C7—C8—C9	120.68 (10)	C17—C18—C19	120.36 (11)
C12—C8—C9	121.42 (10)	C17—C18—H18	119.8
C10—C9—C11	110.02 (11)	C19—C18—H18	119.8
C10—C9—C8	111.90 (10)	C20—C19—C18	120.29 (11)
C11—C9—C8	111.44 (10)	C20—C19—H19	119.9
C10—C9—H9	107.8	C18—C19—H19	119.9
C11—C9—H9	107.8	C19—C20—C21	120.93 (11)
C8—C9—H9	107.8	C19—C20—H20	119.5
C9—C10—H10A	109.5	C21—C20—H20	119.5
C9—C10—H10B	109.5	C22—C21—C16	118.47 (10)
H10A—C10—H10B	109.5	C22—C21—C20	122.91 (11)
C9—C10—H10C	109.5	C16—C21—C20	118.62 (10)
H10A—C10—H10C	109.5	C23—C22—C21	121.52 (10)
H10B—C10—H10C	109.5	C23—C22—H22	119.2
C9—C11—H11A	109.5	C21—C22—H22	119.2
C9—C11—H11B	109.5	C22—C23—C14	120.27 (10)
H11A—C11—H11B	109.5	C22—C23—H23	119.9
C9—C11—H11C	109.5	C14—C23—H23	119.9
H11A—C11—H11C	109.5		
			
O1—C2—C3—C4	12.04 (18)	C3—C2—C14—C23	25.99 (16)
C14—C2—C3—C4	−170.07 (10)	C23—C14—C15—C16	−0.19 (17)
C2—C3—C4—C5	−171.37 (10)	C2—C14—C15—C16	−179.57 (10)
C3—C4—C5—C13	−171.33 (11)	C14—C15—C16—C17	−178.21 (10)
C3—C4—C5—C6	12.27 (18)	C14—C15—C16—C21	1.62 (16)
C13—C5—C6—C7	−2.28 (16)	C15—C16—C17—C18	−179.72 (11)
C4—C5—C6—C7	174.15 (10)	C21—C16—C17—C18	0.46 (17)
C5—C6—C7—C8	0.81 (17)	C16—C17—C18—C19	−0.18 (18)
C6—C7—C8—C12	1.52 (17)	C17—C18—C19—C20	0.11 (19)
C6—C7—C8—C9	−177.39 (10)	C18—C19—C20—C21	−0.32 (19)
C7—C8—C9—C10	121.61 (13)	C17—C16—C21—C22	178.59 (10)
C12—C8—C9—C10	−57.26 (15)	C15—C16—C21—C22	−1.24 (15)
C7—C8—C9—C11	−114.72 (12)	C17—C16—C21—C20	−0.65 (16)
C12—C8—C9—C11	66.40 (15)	C15—C16—C21—C20	179.52 (10)
C7—C8—C12—C13	−2.40 (18)	C19—C20—C21—C22	−178.61 (11)
C9—C8—C12—C13	176.50 (11)	C19—C20—C21—C16	0.60 (17)
C8—C12—C13—C5	0.94 (18)	C16—C21—C22—C23	−0.55 (17)
C6—C5—C13—C12	1.41 (16)	C20—C21—C22—C23	178.65 (11)
C4—C5—C13—C12	−175.14 (10)	C21—C22—C23—C14	2.02 (17)
O1—C2—C14—C15	23.27 (16)	C15—C14—C23—C22	−1.64 (17)
C3—C2—C14—C15	−154.66 (10)	C2—C14—C23—C22	177.71 (10)
O1—C2—C14—C23	−156.08 (11)		
