# Supplementary figures and images for: Crystal structure of 3-[4-(1-methyl­eth­yl)phen­yl]-1-(naphthalen-2-yl)prop-2-en-1-one
Source: Acta Crystallogr Sect E Struct Rep Online. 2014 Aug 16;70(Pt 9):o1009–10. doi: 10.1107/S1600536814017528 (PMC4186083; doi:10.1107/S1600536814017528)

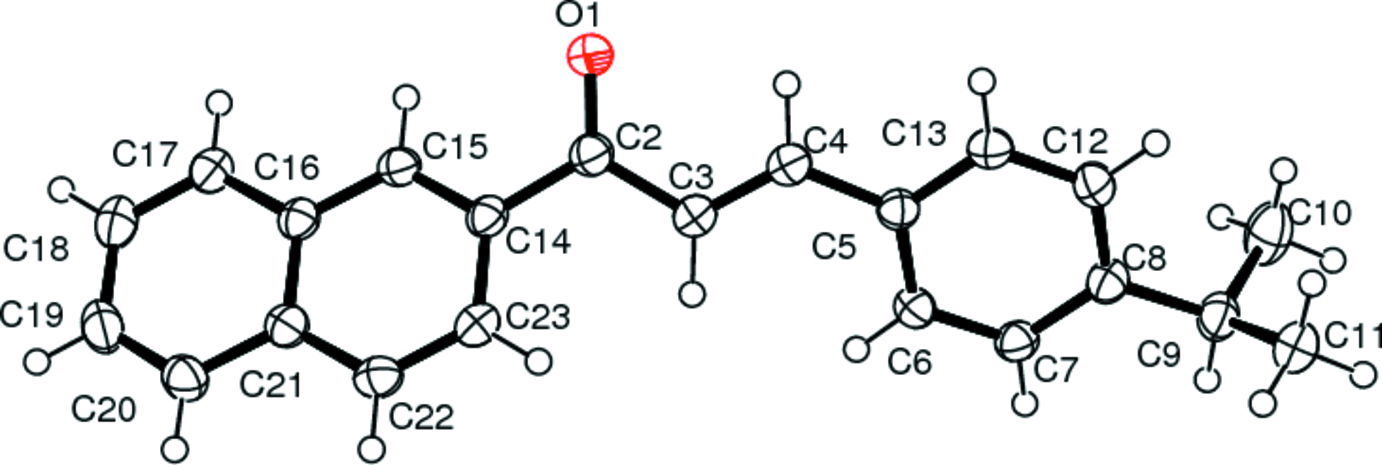

Supplement: Supplementary file 4 [file e-70-o1009-fig1.tif]
